# Synthesis of well-defined bisbenzoin end-functionalized poly(*ε*-caprolactone) macrophotoinitiator by combination of ROP and click chemistry and its use in the synthesis of star copolymers by photoinduced free radical promoted cationic polymerization

**DOI:** 10.1080/15685551.2016.1231041

**Published:** 2016-09-20

**Authors:** Zafer Uyar, Mustafa Degirmenci, Nasrettin Genli, Ayse Yilmaz

**Affiliations:** ^a^ Science Faculty, Department of Chemistry, Harran University, Sanliurfa, Turkey; ^b^ Vocational School of Diyarbakir, Dicle University, Diyarbakir, Turkey

**Keywords:** Ring opening polymerization (ROP), click chemistry, photoinduced free radical promoted cationic polymerization, AB_2_-type miktoarm star copolymer

## Abstract

A new well-defined bisbenzoin group end-functionalized poly(*ε*-caprolactone) macrophotoinitiator (PCL-(PI)_2_) was synthesized by combination of ring opening polymerization (ROP) and click chemistry. The ROP of *ε*-CL monomer in bulk at 110 °C, by means of a hydroxyl functional initiator namely, 3-cyclohexene-1-methanol in conjunction with stannous-2-ethylhexanoate, (Sn(Oct)_2_), yielded a well-defined PCL with a cyclohexene end-chain group (PCL-CH). The bromination and subsequent azidation of the cyclohexene end-chain group gave bisazido functionalized poly(*ε*-caprolactone) (PCL-(N_3_)_2_). Separately, an acetylene functionalized benzoin photoinitiator (PI-alkyne) was synthesized by using benzoin and propargyl bromide. Then the click reaction between PCL-(N_3_)_2_ and PI-alkyne was performed by Cu(I) catalysis. The spectroscopic studies revealed that poly(*ε*-caprolactone) with bisbenzoin photoactive functional group at the chain end (PCL-(PI)_2_) with controlled chain length and low-polydispersity was obtained. This PCL-(PI)_2_ macrophotoinitiator was used as a precursor in photoinduced free radical promoted cationic polymerization to synthesize an AB_2_-type miktoarm star copolymer consisting of poly(*ε*-caprolactone) (PCL, as A block) and poly(cyclohexene oxide) (PCHO, as B block), namely PCL(PCHO)_2_.

## Introduction

Synthesis of novel polymeric materials with improved properties and applications is still an intense and challenging field in polymer chemistry. Among these materials, macrophotoinitiators, defined as polymer systems containing end-chain or mid-chain photoreactive groups, have received great interest in recent years, since they combine the properties of polymers with those of low molecular weight photoinitiators. They have a significant number of advantages in comparison with their corresponding low molecular weight analogs.[1–3] The advantages expected from macrophotoinitiators are better solubility and compatibility within the formulations, low odor, non-toxicity, higher efficiency, and reduced migration to the film surface. Moreover, macrophotoinitiators can be used as prepolymers in photoinitiating systems for synthesizing block and graft copolymers,[4,5] and they also allow combination of polymerizations proceeding with different mechanisms to synthesize polymers with blocks differing in nature.

There have been many publications about the synthesis and characterization of macrophotoinitiators containing photochoromophoric groups in their chains in the literature.[1,6–9] Conventional methods are used in most of these studies. The works related to macrophotoinitiators with well-defined structures are rare though. The discovery of modern synthetic methods has paved the way for new opportunities in the preparation of well-defined macrophotoinitiators with controlled molecular weight and lower polydispersity. Controlled/living polymerization methods such as atom transfer radical polymerization (ATRP) and ring opening polymerization (ROP) can be employed for this purpose. There are two procedures offered to synthesize well-defined macrophotoinitiators by using these methods: (i) the use of a photoinitiator with suitable functionality as an initiator in ATRP or ROP of a monomer system [10–13]; (ii) functionalization of a polymer achieved by ATRP or ROP with a photoreactive group.[14–17]

We have previously synthesized and characterized many well-defined macrophotoinitiators of poly(*ε*-caprolactone) (PCL),[10,14,16] polystyrene (PSt),[18–21] and Poly(methyl methacrylate) (PMMA) [12] with various photoactive functionalities by using above methods and used them as prepolymers in the synthesis of block copolymers. To the best of our knowledge, very few works about the well-defined polymers having benzoin moiety have been reported. In preceding papers,[10,11,22] we applied ATRP and ROP methods to incorporate benzoin photoinitiator moiety to the chain end of the polymers. Thus, polystyrenes and PCLs with benzoin end-functionalities and narrow molecular weight distributions were prepared by using respective functional initiators in the ATRP and ROP systems. We have recently synthesized benzoin functionalized well-defined telechelic macrophotoinitiator of polystyrene by a combination of ATRP and click chemistry, which has a potential in initiating light-induced free radical promoted cationic polymerization to obtain ABA type tri-block copolymer.[15] Quite recently, we have also applied a combination of ROP and click chemistry in order to synthesize well-defined benzoin end-functionalized PCL.[16] This macrophotoinitiator was used as a prepolymer in photoinduced polymerzation for achieving AB type di-block copolymer. In this paper we have synthesised a well-defined bisbenzoin end-group photoactive functionalized poly(*ε*-caprolactone) (PCL-(PI)_2_) by combination of ROP and click chemistry. In contrast to our previous studies, the obtained macrophotoinitiator here was used as precursor in photoinitiated polymerization to get AB_2_-type miktoarm star copolymer comprising of poly(*ε*-caprolactone) (PCL, as A block) and poly(cyclohexene oxide) (PCHO, as B block).

Star type polymers consisting of several identical (homo-armed star polymers) or unequal linear chains (hetero-armed or miktoarm star copolymers) together have received great interest owing to their distinctive morphological and physical properties and possible processing advantages resulting from their unique architectures.[23–26] Miktoarm star copolymers represent a structure in which polymer blocks are connected to one another at a central core. There are many routes to synthesize such kind of polymers. The most common and well established route to synthesize such polymers mainly contains two steps. In the first step, a linear polymer with controlled chain length and end-groups is prepared by a controlled/living polymerization method such as ATRP or ROP. Then, the end-groups are converted to suitable chemical functionalities which undergo a facile and specific reaction (usually a ‘click’) to build the desired structure.[27,28] Various types of miktoarm star copolymers such as AB_2_,[29,30] A_2_B,[23] A_2_B_2_,[31] AB_3_,[28,29] ABC[32] … etc., have been synthesized and characterized by using this way.

In this study, we report a novel well-defined bisbenzoin group end-functionalized poly(*ε*-caprolactone) macrophotoinitiator (PCL-(PI)_2_) via the combination of ROP and ‘click chemistry’ methods. This macrophotoinitiator was then used as a prepolymer in the synthesis of an AB_2_-type miktoarm star copolymer comprising of PCL (as A block) and PCHO (as B block) by photoinduced free radical promoted cationic copolymerization. To the best of our knowledge, the synthesis of bisbenzoin group end-functionalized PCL macrophotoinitiator and AB_2_ miktoarm star copolymer with linear PCL and PCHO as building blocks has not been reported.

## Experimental

### Materials

*ε*-Caprolactone (*ε*-CL) (Aldrich) and Cyclohexene oxide (CHO) (Aldrich) were distilled over calcium hyride (CaH_2_) and stored in a refrigerator under nitrogen before use. Acetylene-functionalized benzoin (PI-alkyne) was synthesized by slightly modifying the procedure described in the literature.[15] Stannous-2-ethylhexanoate (Sn(Oct)_2_) (Aldrich), 3-cyclohexene-1-methanol (Aldrich), Sodium azide (Aldrich), Propargyl bromide (80 wt. % in toluene, Aldrich), Diphenyliodonium hexafluorophosphate (Fluka), CuBr (Aldrich), Iodic acid (HIO_3_) (Sigma-Aldrich), Potassium bromide (KBr) (Sigma-Aldrich), Sodium thiosulfate (Na_2_S_2_O_3_) (Sigma-Aldrich), Sodium azide (NaN_3_) (Merck), Tetrabutylammonium bromide (Sigma-Aldrich), and 2,2*ꞌ*-bipyridine (Merck) were used as received. 1-Ethoxy-2-methylpyridinium hexafluorophosphate (EMP^+^PF_6_^−^) was prepared according to the published procedure.[33] Solvents, dichloromethane (CH_2_Cl_2_), toluene, and tetrahydrofuran (THF), were distilled over drying agents under nitrogen prior to use.

### Synthesis of cyclohexene end-functionalized poly(*ε*-caprolactone) (PCL-CH)

Cyclohexene end-functionalized poly(*ε*-caprolactone) (PCL-CH) was obtained according to the method previously described in the literature [34] by ROP of *ε*-CL using Sn(Oct)_2_ as catalyst and 3-cyclohexene-1-methanol as the initiator. Typically, 3-cyclohexene-1-methanol (253 mg, 2.25 mmol), *ε*-caprolactone (5.15 g, 45 mmol), and stannous-2-ethylhexanoate (2.30 mg, 5.63 × 10^−3^ mmol) were placed under nitrogen in a previously flamed and nitrogen-purged schlenk flask equipped with magnetic stirrer bead. The reaction mixture was degassed by freeze-thaw cycles (×3). The flask was charged with N_2_ and the reaction mixture was allowed to heat up to 110 °C. After 5 days of stirring, the reaction was allowed to cool at room temperature. Upon cooling, a solid polymer formation was observed. The resultant solid was dissolved in dichloromethane to give a viscous solution. This viscous solution was added drop-wise to 200 ml of cold methanol by stirring and the precipitated polymer was then filtered off using a Gooch crucible, washed with cold methanol and dried under vacuum at room temperature to obtain PCL-CH. Yield: 4.89 g, 95%. *M*_n H NMR_ = 2300, *M*_n GPC_ = 2500, *M*_w_/*M*_n_ = 1.22.

### Bromination of cyclohexene end-functionalized poly(*ε*-caprolactone) (PCL-(Br)_2_)

Cyclohexene end-functionalized poly(*ε*-caprolactone) (PCL-CH) (4 g, 2.93 mmol) was dissolved in 50 mL of dichloromethane in a two-necked round-bottomed flask. A solution of KBr (1.05 g, 8.7 mmol) in a 50 mL of distilled H_2_O was added to the reaction flask. Finally, HIO_3_ (1.8 g, 10.23 mmol) was added to the reaction mixture and the reaction was stirred at room temperature. At first, upper water phase was yellowish and bottom dichloromethane phase was colorless. After 5 min., bottom phase turned to orange. The reaction progress was monitored by TLC. After 24 h of stirring, the reaction was worked up by addition of 100 mL of 10% Na_2_S_2_O_3_ solution. This mixture was transferred into a separatory funnel and the organic layer was extracted with dichloromethane (3 × 70 mL) and dried over anhydrous Na_2_SO_4_ and filtered. Evaporation of the excess solvent gave a grayish solid which was dissolved in dichloromethane to give a viscous solution and purified by reprecipitation from cold methanol. Dibromocyclohexane end-functionalized poly(*ε*-caprolactone) (PCL-(Br)_2_) was collected after filtration and dried at room temperature in a vacuum for 3 days. Yield: ~4.30 g, >99%. *M*_n H NMR_ = 2510, *M*_n GPC_ = 2900, Mw/*M*_n_ = 1.24.

### Azidation of dibromocyclohexane end-functionalized poly(*ε*-caprolactone) (PCL-(N_3_)_2_)

A suspension of dibromocyclohexane end-functionalized poly(*ε*-caprolactone) (PCL-(Br)_2_) (3.50 g, ~2.30 mmol) and NaN_3_ (0.89 g, 13.75 mmol) in 20 ml of dimethylformamide (DMF) was stirred at room temperature for 24 h in a 100 mL round-bottomed flask. Undissolved substance (NaN_3_) was removed by filtration through filter paper. The clear solution obtained was concentrated under vacuum and the resultant solution was added drop-wise to a cold mixture of 200 ml methanol and 50 ml water by stirring. The white polymeric compound, diazidocyclohexane end-functionalized poly(*ε*-caprolactone) (PCL-(N_3_)_2_), was then filtered off using a Gooch crucible, washed with cold methanol-water mixture and dried under vacuum at room temperature for 3 days. Yield: ~3.50 g, >99%. *M*_n H NMR_ = 2480, *M*_n GPC_ = 3000, Mw/*M*_n_ = 1.25.

### Synthesis of acetylene end-functionalized benzoin (PI-alkyne)

Acetylene end-functional benzoin namely, 1,2-diphenyl-2-(propargyloxy)ethanone (PI-alkyne) was synthesized by slightly modifying the procedure described in the literature.[15] A solution of NaOH (2.35 g, 58.75 mmol) in 100 mL of distilled H_2_O was prepared in a 500 mL two-neck round-bottom flask. Benzoin (10 g, 47.00 mmol) was then added to this solution and the reaction was heated to 50 °C. Toluene (dry, 50 mL) was then added under stirring conditions until benzoin was dissolved completely forming a clear solution. The color of the mixture turned to orange-yellow during this process. To this clear solution, tetrabutylammonium bromide (1.89 g, 5.87 mmol) was added as a phase transfer catalyst. A separate solution was prepared by dissolving propargyl bromide (7.00 g, 58.75 mmol) in 50 mL of dry toluene and this solution was added drop wise to the first reaction flask during 30 min. The reaction mixture was kept stirring at 50 °C for 24 h and the reaction progress was monitored by TLC. Due to the presence of some starting material on TLC plate, 5 g NaOH and 100 mL of DMSO were added to the reaction and the mixture was further stirred for another 2 h until TLC analysis showed no starting material. The color was turned to black from orange. This mixture was transferred into a separatory funnel and extracted with toluene (100 mL) and water (100 mL) after complete separation of the phases. The organic layer was dried over anhydrous Na_2_SO_4_ and filtered. The crude product was purified by column chromatography (SiO_2_, CH_2_Cl_2_) to afford acetylene-functionalized benzoin. Yield: 5.40 g, 46%, Rf 0.55.

### Synthesis of bisbenzoin end-functionalized poly(*ε*-caprolactone) macrophotoinitiator by click reaction of PCL-(N_3_)_2_ with PI-alkyne (PCL-(PI)_2_)

Diazide end-capped poly(*ε*-caprolactone) (PCL-(N_3_)_2_) (581 mg), 1,2-diphenyl-2-(prop-2-ynyloxy)ethanone (PI-alkyne) (300 mg, 1.2 mmol), oven-dried CuBr (344 mg, 2.4 mmol) and bipyridine (750 mg, 4.8 mmol) were placed in a 100 mL Schlenk flask and the reagents were further dried under vacuum. After the flask was charged with N_2_, dry THF (20 mL, freshly distilled over Na under N_2_) was added. The reaction mixture was degassed by freeze-thaw cycles (×4) and then stirred at room temperature for 24 h under N_2_. The reaction color which was initially dark brown turned to gray ash color. The solvent was evaporated to dryness. The resultant residue was dissolved in dichloromethane (30 mL) and washed with water (30 ml × 4) in a separatory funnel until blue color of copper catalyst was not detected in water phase. The organic layer was dried over anhydrous Na_2_SO_4_ and filtered. The resulting solution was passed through an alumina column in order to remove trace amount of copper catalyst left from the mixture. After evaporating the excess solvent, the mixture was precipitated in cold methanol. Bisbenzoin end-eunctionalized PCL macrophotoinitiator (PCL-(PI)_2_) was filtered off using a Gooch crucible and dried under vacuum for 3 days. Yield: ~650 mg, >99%, *M*_n H NMR_ = 2810, *M*_n GPC_ = 3200, and Mw/*M*_n_ = 1.24.

### Synthesis of AB_2_-type miktoarm star copolymer by free radical promoted cationic polymerization (PCL(PCHO)_2_)

A certain amount of the macrophotoinitiator (PCL-(PI)_2_) and the monomer (cyclohexene oxide, CHO) in bulk containing onium salt (Ph_2_I^+^ or EMP^+^) in Pyrex tubes were degassed with nitrogen prior to irradiation. At the end of irradiation in a merry-go-round type photoreactor equipped with 16 Philips 8 W/08 lamps emitting light nominally at 350 nm at room temperature, the solutions were poured into cold methanol. The precipitated AB_2_-type miktoarm star copolymers were filtered off and dried in vacuo.

### Characterization

^1^H NMR spectra were recorded on an Agilent 400 MHz NMR spectrometer at room temperature. Fourier transform infrared (FT-IR) spectra were measured with a Perkin-Elmer Spectrum Two FT-IR spectrophotometer. Number and weight average molecular weights (*M*_n_ and *M*_w_) and molecular weight distributions (*M*_w_/*M*_n_) were determined by gel permeation chromatography (GPC) using an Agilent GPC Instrument (Model 1100) consisting of a pump, a refractive index detector and two Waters Styragel columns (HR 5E, HR 4E), using THF as the eluent at a flow rate of 0.5 mL/min at 23 °C and toluene as an internal standard. Molecular weights were calculated by using monodisperse polystyrene standards. UV–vis spectra were registered on a Schimadzu 1601 spectrophotometer. Fluorescence spectra were recorded on a Shimadzu RF-1501 spectrofluorophotometer. Thermal stabilities and the glass transition temperatures of the polymers were investigated on a Perkin-Elmer TGA/DTA 7300 thermal analysis systems, under N_2_ flow with a heating rate of 20 °C/min. Molecular weights of PCL-CH, PCL-(Br)_2_, PCL-(N_3_)_2_ and PCL-(PI)_2_ were calculated with the aid of polystyrene standards by using the following conversion formula [35]: *M*_PCL_ = 0.259 *M*_PSt_^1.073^.

## Results and discussion

### Synthesis of cyclohexene end-functionalized poly(*ε*-caprolactone) (PCL-CH)

**Scheme 1.**
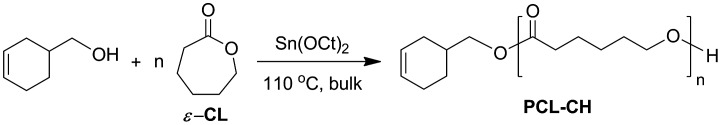
Synthesis of PCL-CH via ROP.

The synthesis of cyclohexene end-functionalized poly(*ε*-caprolactone) (PCL-CH) was performed [34] by ROP of *ε*-CL using Sn(Oct)_2_ as catalyst and 3-cyclohexene-1-methanol as the initiator. The molar ratios of [*ε*-CL]/[3-cyclohexene-1-methanol] and [3-cyclohexene-1-methanol]/[Sn(Oct)_2_] were taken as 20/1 and 400/1, respectively. With this initiator system, PCL functionalized with cyclohexene end-group has been obtained as depicted in Scheme 1.

**Figure 1.**
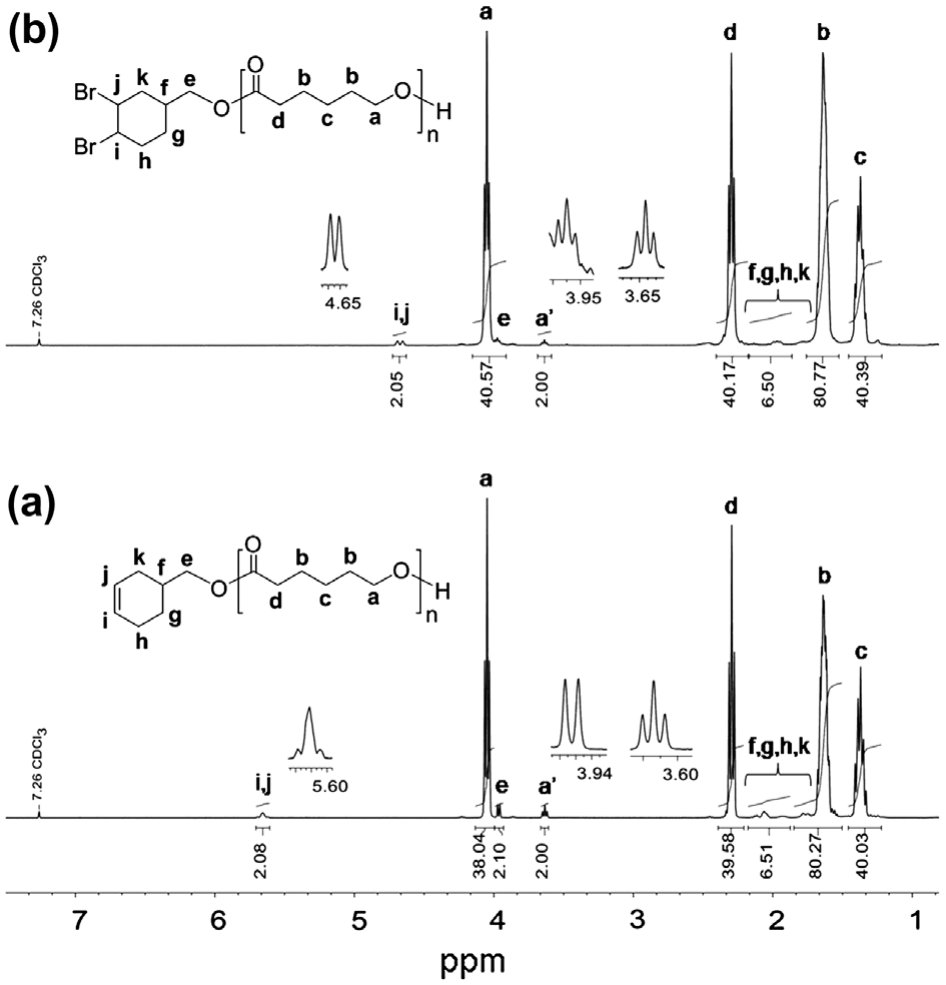
^1^H NMR spectra of PCL-CH (a) and PCL-(Br)_2_ (b) in CDCl_3_.

The ^1^H NMR spectrum of PCL-CH showed the resonance signals of the double bond protons of cyclohexene ring (at the PCL end-chain) at 5.71–5.60 ppm, the −CH_2_−O−CO protons at 3.97 and 3.95 ppm, the repeating-unit protons of PCL at 4.06–4.03, 2.33–2.27, 1.71–1.58, and 1.42–1.31 ppm, and the signals assignable to the methylene protons adjacent to the ω-chain end hydroxyl groups (proton a′) were clearly observed at 3.65–3.62 ppm, which indicated the completion of ROP (Figure 1(a)). The GPC measurements of PCL-CH indicated unimodal and narrow GPC trace with *M*_n GPC_ = 2500 (Figure 3(a)) and *M*_w_/*M*_n_ = 1.22. In addition, the calculated *M*_n_ (*M*_n theo_ = 2280) and measured *M*_n_’s (*M*_n GPC_ = 2500 and *M*_n H NMR_ = 2300) are in good agreement indicating the formation of well-defined cyclohexene end-functionalized PCL. The experimental conditions and results are summarized in Table 1.

**Figure 2.**
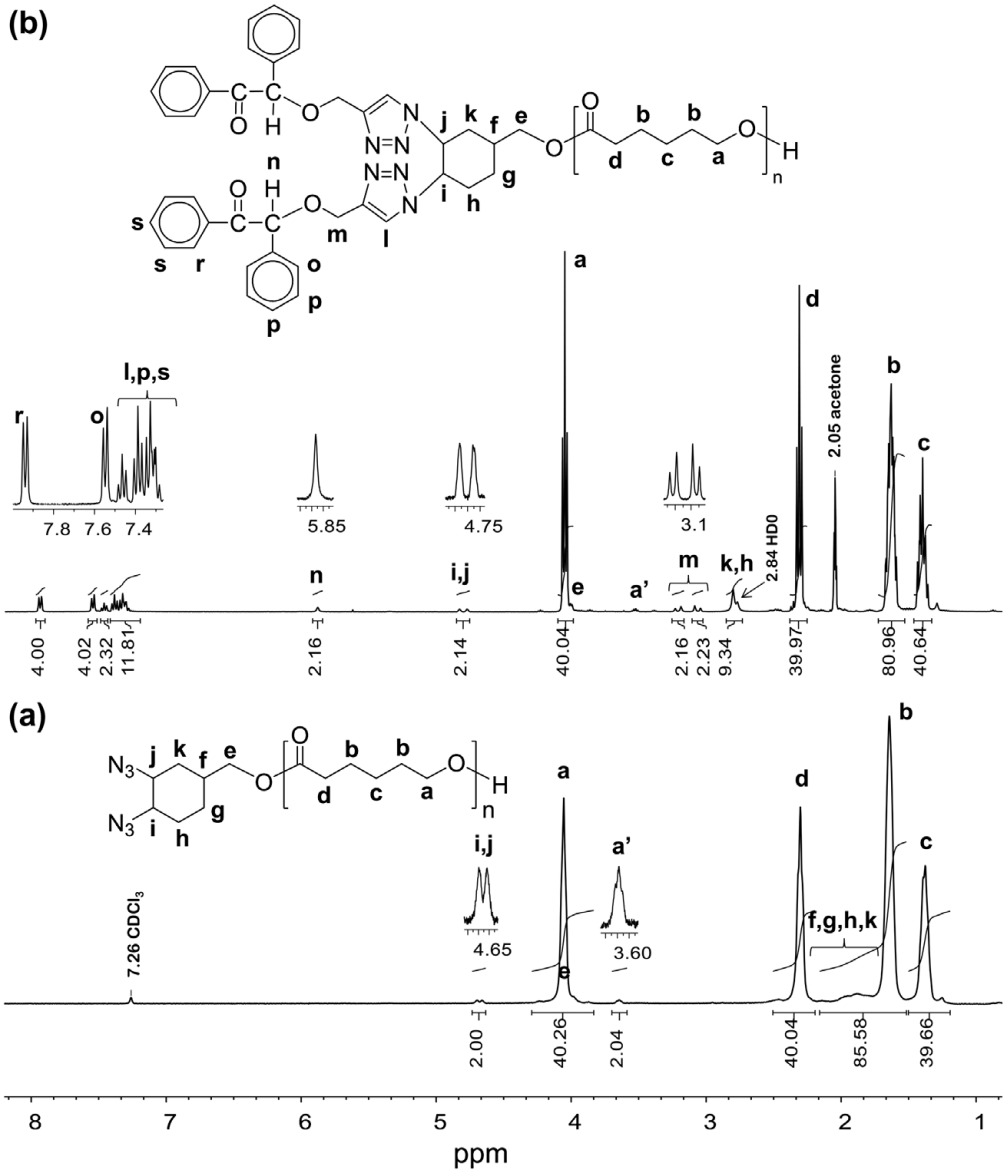
^1^H NMR spectra of PCL-(N_3_)_2_ (a) and PCL-(PI)_2_ (b) in deuterated acetone.

**Figure 3.**
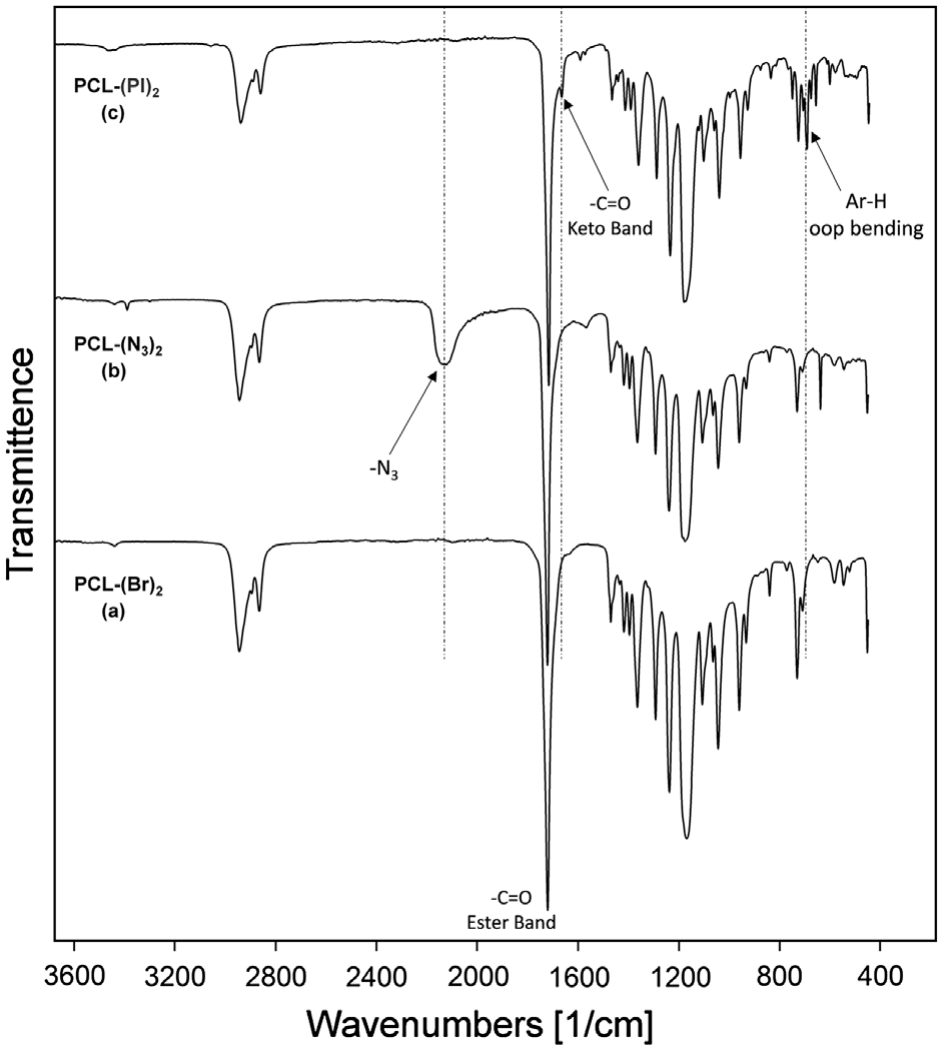
FT-IR spectra of PCL-(Br)_2_ (a), PCL-(N_3_)_2_ (b) and PCL-(PI)_2_ (c).

**Figure 4.**
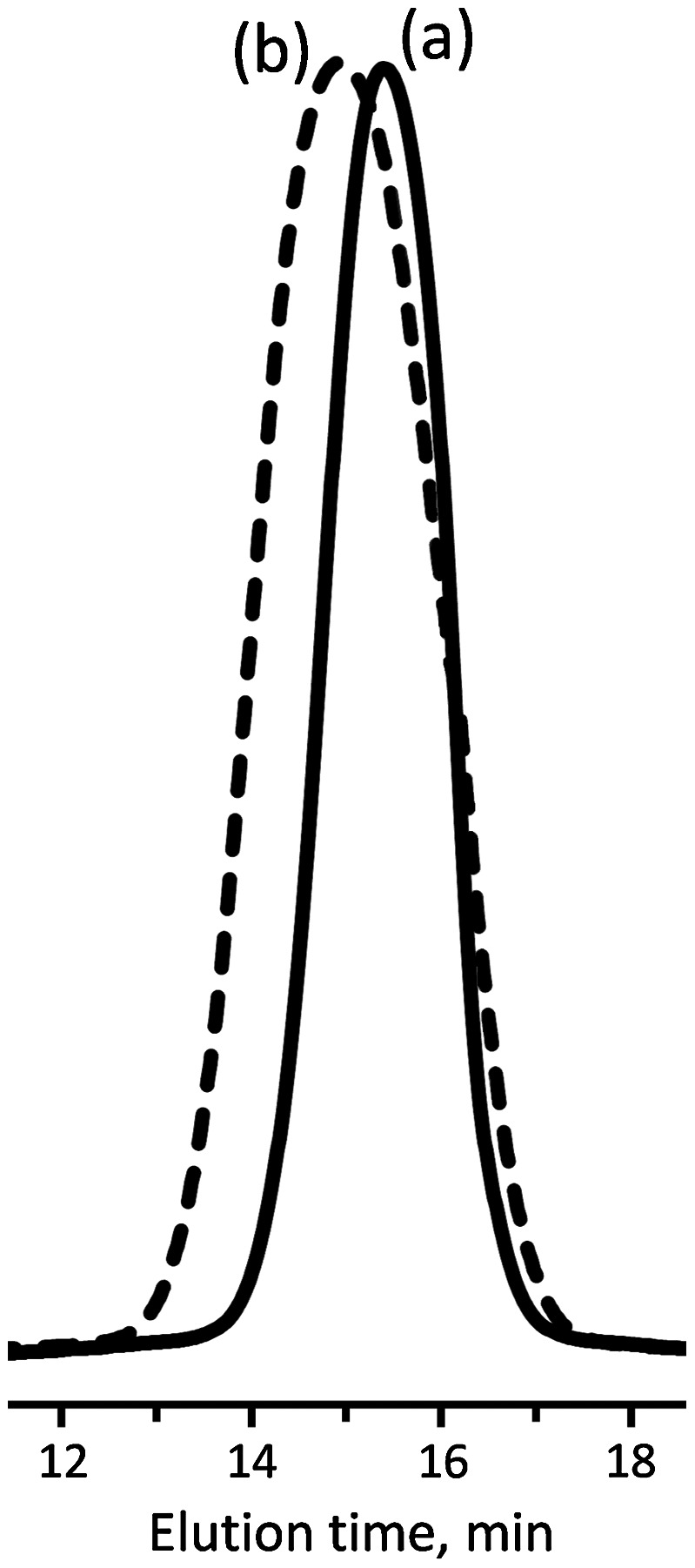
GPC traces of PCL-(PI)_2_ (a) and PCL(PCHO)_2_ (b).

**Figure 5.**
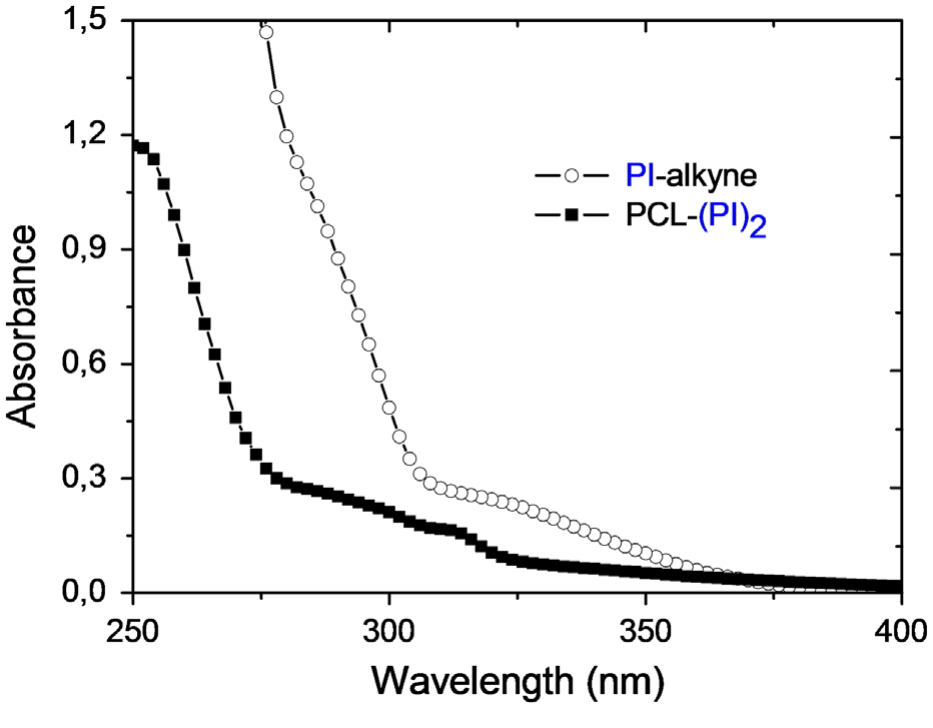
UV absorption spectra of PI-alkyne (6.15 × 10^−4^ mol L^−1^) and PCL-(PI)_2_ (0.16 g L^−1^) in CH_2_Cl_2_.

**Figure 6.**
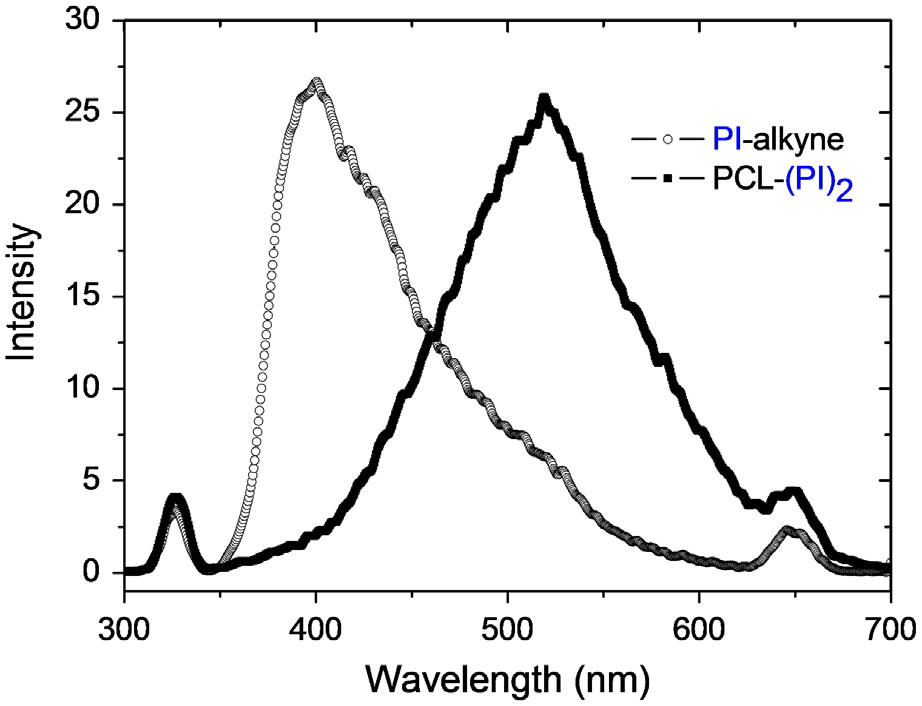
Fluorescence spectra of PI-alkyne (1.60 × 10^−2^ mol L^−1^) and PCL-(PI)_2_ (4.00 g L^−1^) in CH_2_Cl_2_, *λ*_exc_ = 325 nm.

**Figure 7.**
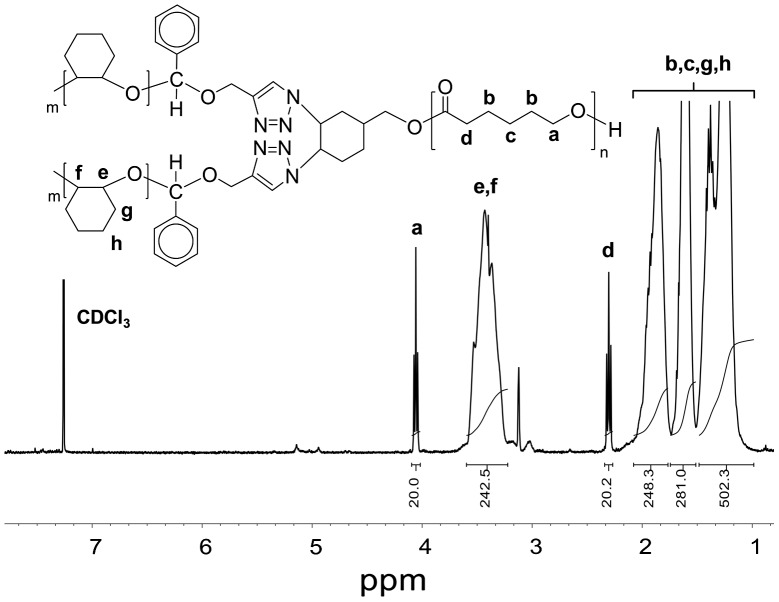
^1^H NMR spectra of PCL(PCHO)_2_ miktoarm star type copolymer in CDCl_3_.

**Figure 8.**
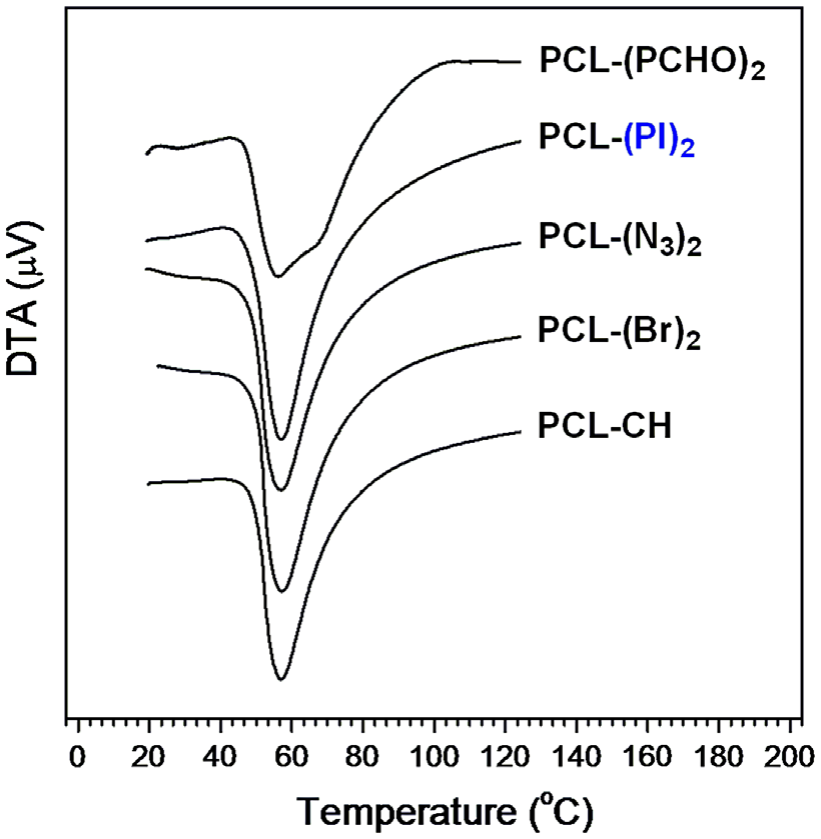
DTA traces of synthesized polymers.

**Figure 9.**
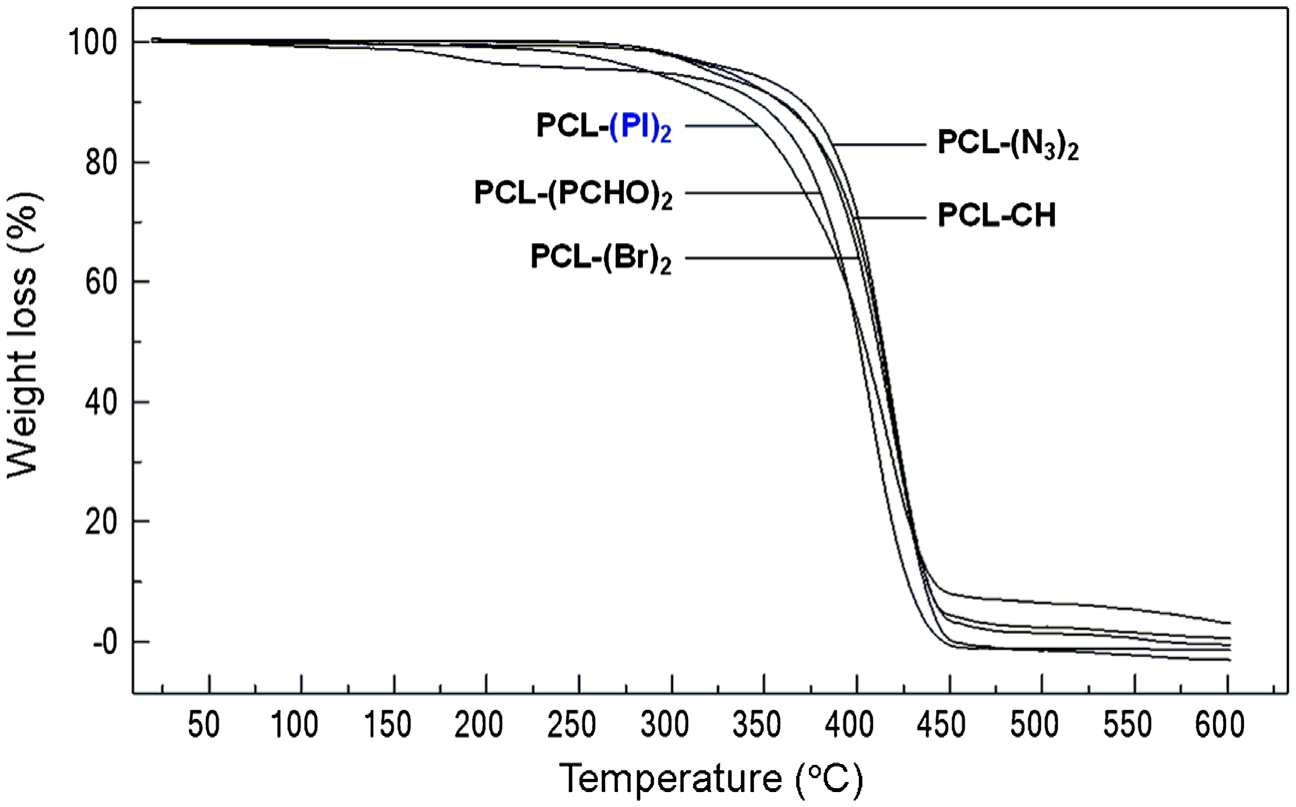
TGA thermograms of synthesized polymers.

**Table 1.** Conditions and results of the PCL-CH^a^, PCL-(Br)_2_ ^b^, PCL-(N_3_)_2_ ^c^ and PCL-(PI)_2_ ^d^.

Run	Polymer	Temp. (°C)	Conversion (%)	*M*_n theo_	*M*_n H NMR_	*M*_n.GPC_^e^	*M*_w_/*M*_n_
1	PCL-CH	110	95	2280	2300	2500	1.22
2	PCL-(Br)_2_	25	>99	2440	2510	2900	1.24
3	PCL-(N_3_)_2_	25	>99	2365	2480	3000	1.25
4	PCL-(PI)_2_	25	>99	2865	2810	3200	1.24

^a^ Prepared by ROP of *ε*-CL. [*ε*-CL]_0_ = 9.02 mol L^−1^(in bulk), [I]/[ [*ε*-CL] = 1/20 and [Sn(Oct)_2_]/[I] = 1/400, 110 °C.

^b^ Prepared via bromination of PCL-CH.

^c^ Prepared via azidation of PCL-(Br)_2._

^d^ Prepared via ‘click’ reaction between PCL-(N_3_)_2_ and PI-alkyne.

^e^ Determined by GPC according PSt standards.

### Synthesis of dibromo- and diazido-cyclohexane end-functionalized poly(*ε*-caprolactone) [(PCL-(Br)_2_) and (PCL-(N_3_)_2_)]

**Scheme 2.**
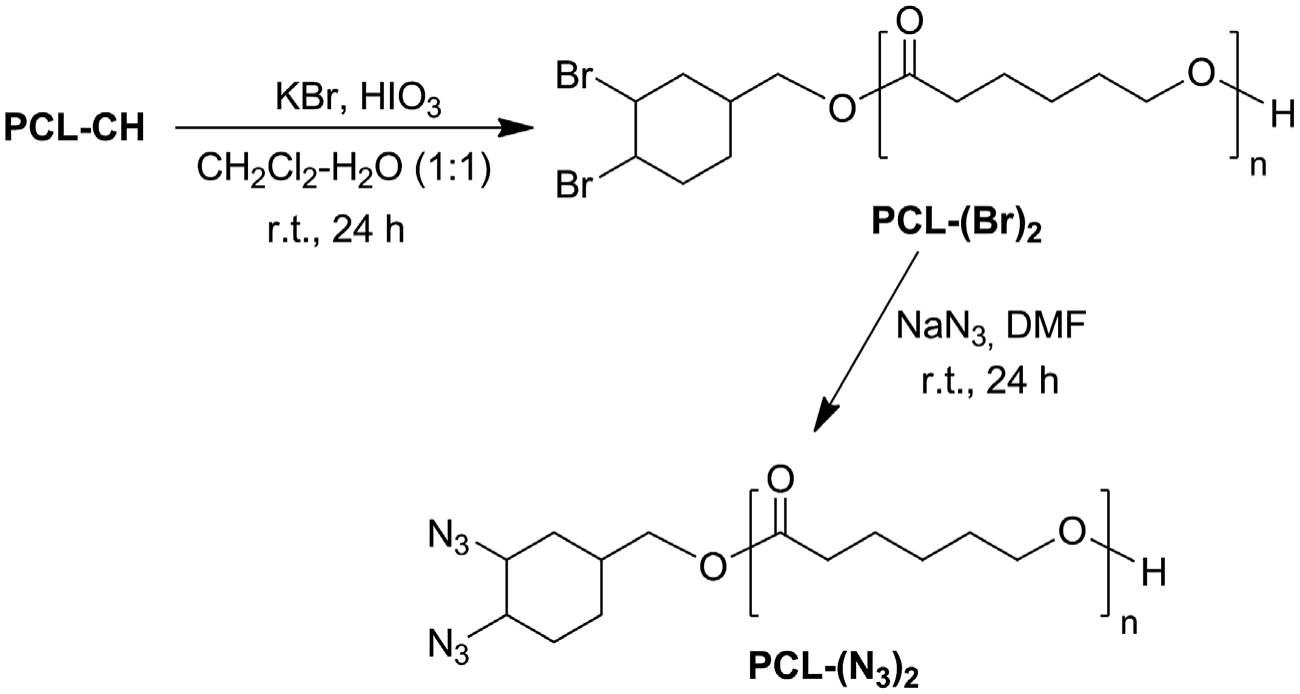
Synthetic procedures for PCL-(Br)_2_ and PCL-(N_3_)_2_.

Bromination of cyclohexene end-functionalized group of PCL was conducted using potassium bromide (KBr) and iodic acid (HIO_3_) in a two-phase system consisting of CH_2_Cl_2_/H_2_O (1/1) at room temperature.[36] Dibromo-cyclohexane end-functionalized PCL was obtained in ~99% yield. Subsequently, the bromines at the chain ends of PCL were transformed into azido groups through the simple nucleophilic substitution reaction with NaN_3_ in DMF. The overall reaction for bromination and azidation of PCL is shown in Scheme 2.

The dibromo- and diazido-cyclohexane end-functionalized PCL precursors were identified by ^1^H NMR, FT-IR, and GPC measurements. Figure 1(b) shows the ^1^H NMR spectrum of the prepolymer obtained from the bromination of PCL-CH. After bromination, the characteristic signals at 5.71–5.60 ppm for double bond protons of cyclohexene end group (in Figure 1(a)) completely disappeared, and the new resonance signals that appeared at 4.70 and 4.66 ppm are ascribed to protons of CH-Br (Figure 1(b)). This polymer has also showed the signals from the PCL backbone at 4.07–4.04 ppm (proton a), 2.32–2.28 ppm (proton d), 1.69−1.55 ppm (protons b), and 1.42–1.32 ppm (proton c) along with the signals that originated from the corresponding initiator residues. The ^1^H NMR spectrum of the diazido end-functionalized prepolymer is depicted in Figure 2(a). The good agreement between the molecular weight distributions (PDI) and the calculated and measured *M*_n_’s of PCL polymers (which are summarized in Table 1) indicates that no side reactions occurred during the bromination and nucleophilic substitution reaction (azidation) and well-defined dibromo and diazido end-functionalized PCLs were obtained. The structures of PCL-(Br)_2_ and PCL(N_3_)_2_ were further supported by the observations from IR spectra as indicated in Figure 3(a) and (b). The IR measurements provided direct evidence for the presence of azide units with a typical vibration band at 2120 cm^−1^ which was absent in the case of PCL-(Br)_2_.

### Synthesis of bisbenzoin end-functionalized poly(*ε*-caprolactone) macrophotoinitiator (PCL-(PI)_2_)

**Scheme 3.**
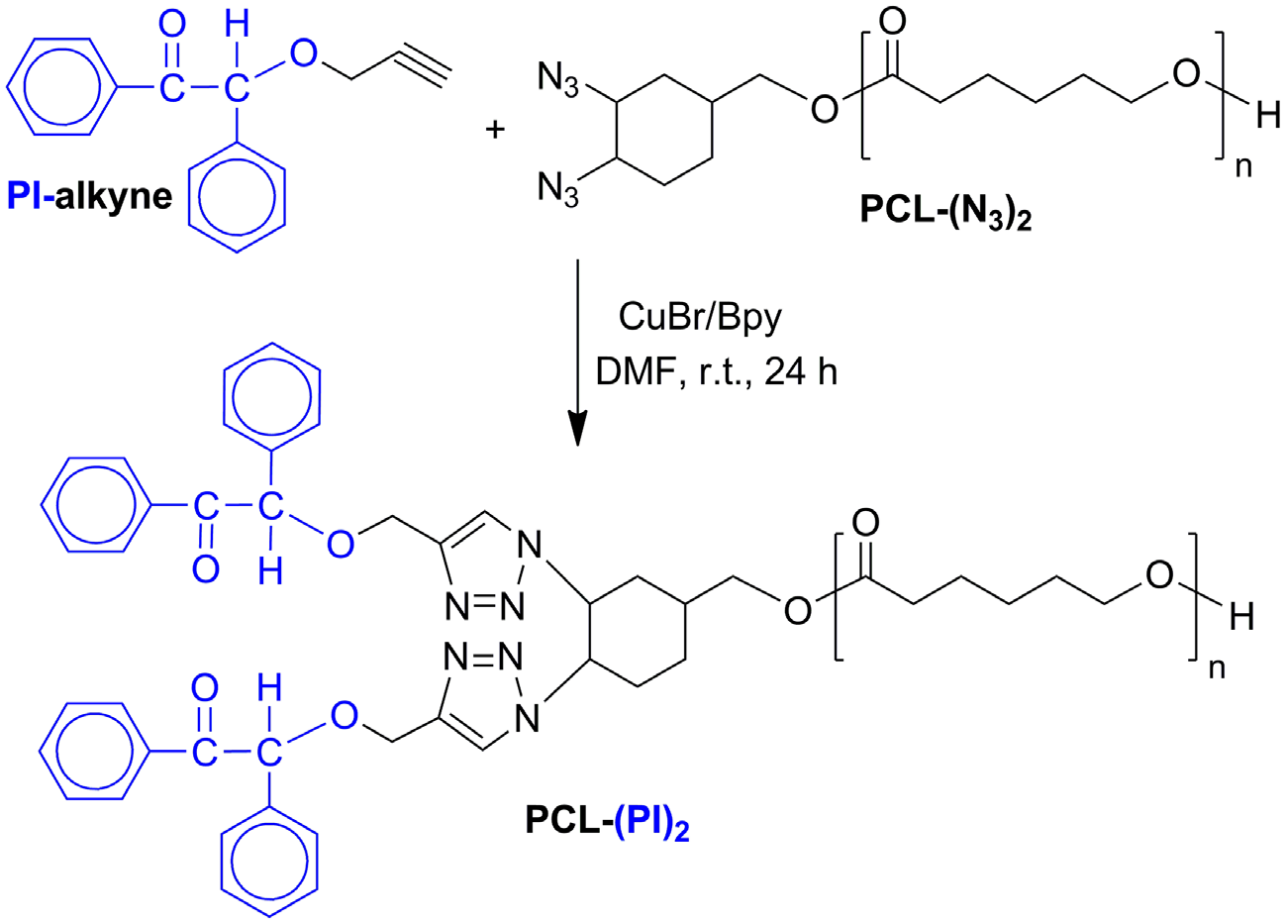
Synthesis of bisbenzoin end-functionalized poly(*ε*-caprolactone) macrophotoinitiator, PCL-(PI)_2_, via click chemistry.

The copper-catalyzed azide-alkyne cycloaddition reaction (click reaction) was employed for the preparation of (PCL-(PI)_2_). The acetylene-functionalized benzoin (PI-alkyne), which is a click couple compound, was synthesized by slightly modifying the procedure described previously with 46% yield. The click reaction of PCL(N_3_)_2_ with PI-alkyne was performed in DMF at room temperature for 24 h using the CuBr/bipyridine catalyst system, as shown in Scheme 3. Polymer solution was passed through neutral alumina column to remove copper salt and the resin, then the solvent was removed by a rotary evaporator and the product was obtained as white solid from precipitation in methanol.

The FT-IR spectrum of the PCL-(PI)_2_ (Figure 3(c)) indicated the disappearance of the absorption peak of the azido groups at around 2120 cm^−1^ suggesting that there is no unreacted azide functionalized polymer in the reaction products. In addition, the IR spectrum of the ‘click’ coupled product (PCL-(PI)_2_) contains the characteristic C=O ester band of PCL chain at 1720 cm^−1^, the C=O keto group of the photoinitiator moiety at 1670 cm^−1^, and the oop bending of Ar–H from benzoin end groups at 697 cm^−1^ demonstrating quantitative coupling and the successful formation of PCL-(PI)_2._ Further evidence for the formation macrophotoinitiator could be seen in the ^1^H NMR spectrum in Figure 2(b). The signals due to benzoin aromatic protons at the end-groups of the PCL-(PI)_2_ at 7.88 and 8.06 ppm were observed in this spectrum along with the characteristic signals due to the repeating units of the macrophotoinitiator itself. The GPC trace of the PCL-(PI)_2_ macrophotoinitiator shown in Figure 4(a) displayed a unimodal peak and a little shifted toward the higher molecular weight region as compared to the starting material. In addition, its molecular weight distribution is still low and the theoretical, GPC and ^1^H NMR molecular weights of the PCL-(PI)_2_ are fit with each other indicating the formation of the macrophotoinitiator with well-defined structure.

Even more convincing evidences for the incorporation of benzoin groups at the end-chain of PCL-(PI)_2_ macrophotoinitiator and the completion of click cyclization reaction were obtained from UV–vis and Fluorescence measurements. Figure 5 shows the absorption spectra of PCL-(PI)_2_ together with the PI-alkyne synthesized. Each spectrum has specific absorptions between 300 and 400 nm. It can be clearly seen that the polymeric photoinitiator, PCL-(PI)_2_, has spectrum which is typical for benzoyl chromophores absorbing strongly in far UV and possessing an absorption maximum of the n→π* transitions around 320 nm (*λ*_max_ = 320 nm, *ε* = 100–200 L mol^−1^ cm^−1^).[37] Thus these types of polymeric photoinitiators are suitable for use in formulations which do not absorb strongly in this region.

Figure 6 shows the fluorescence emission of the PCL-(PI)_2_ macrophotoinitiator and its precursor PI-alkyne in CH_2_Cl_2_ at room temperature. All spectra show the vibrational structures of the phenyl ketone chromophore. All these spectroscopic investigations suggest that the photochromophoric benzoin groups were conserved under the click cyclization reaction conditions.

### Synthesis of AB_2_-type miktoarm star copolymer (PCL(PCHO)_2_)

**Scheme 4.**
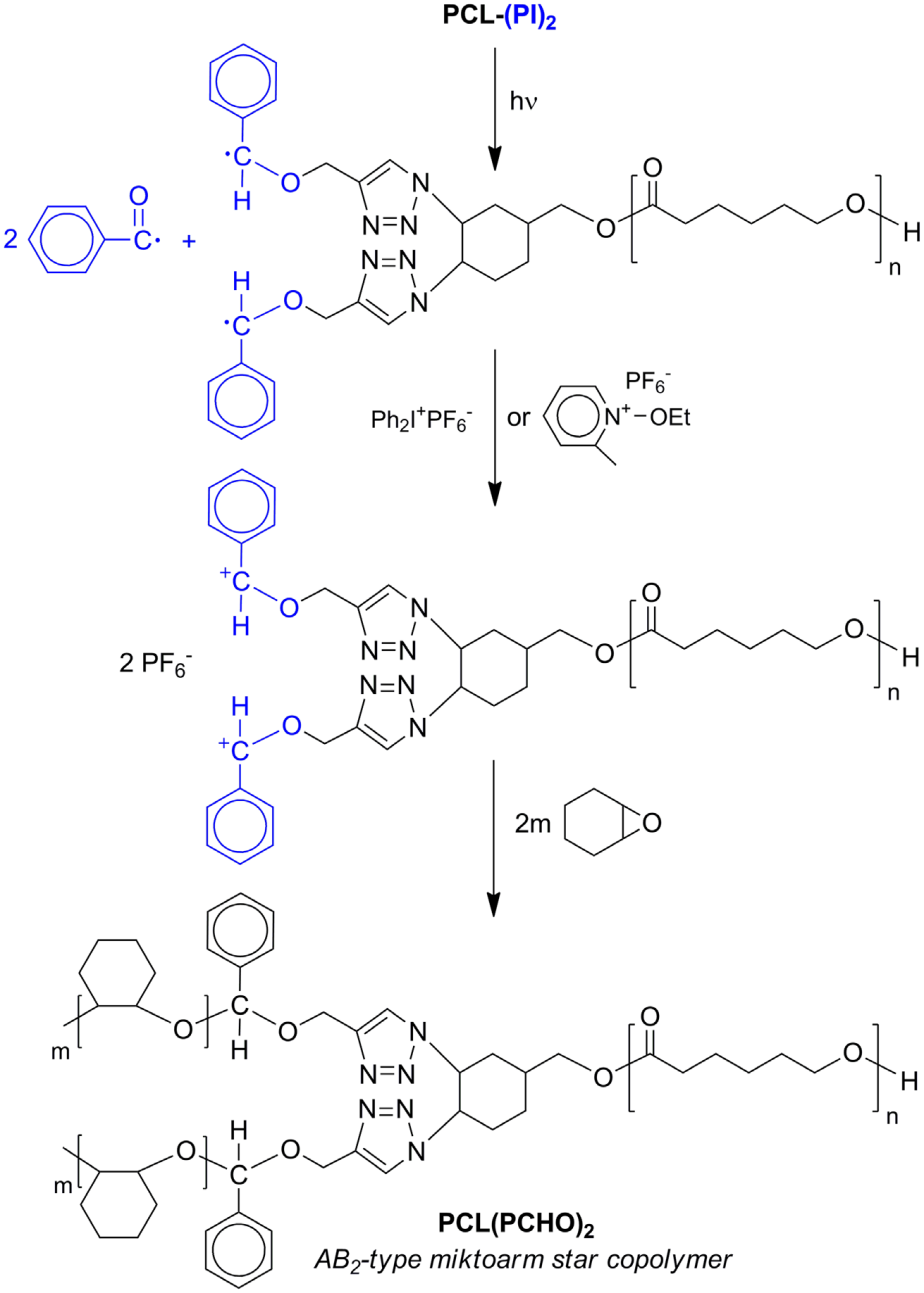
Synthesis of AB_2_-type miktoarm star copolymer, PCL(PCHO)_2_, via photoinitiated free radical promoted cationic polymerization method.

The photoinitiated free radical promoted cationic polymerization method was employed for the preparation of PCL(PCHO)_2_ (AB_2_-type miktoarm star copolymer) by using PCL-(PI)_2_ macrophotoinitiator as the photoinitiator and CHO as the monomer. The initial stage of the UV irradiation process is radicalic. UV irradiation of the PCL-(PI)_2_ macrophotoinitiator caused α-scission and yielded benzoyl (electron withdrawing), and polymer bound radicals (electron donating), according to the following reaction (Scheme 4). It is known that certain onium salts such as Ph_2_^+^PF_6_^−^ and EMP^+^PF_6_^−^ can efficiently oxidize photochemically generated electron donating free radicals.[22] If the photolysis is carried out in the presence of a cationic polymerizable monomer such as CHO the polymer attached radicals are converted to initiating cations to generate AB_2_-type miktoarm star copolymer consisting of poly(*ε*-caprolactone) (PCL, as A block) and poly(cyclohexene oxide) (PCHO, as B blocks). It should be pointed out that benzoyl radicals formed concomitantly do not participate in the oxidation process.[38] In addition, no peak belonging to the homopolymer formation was observed on GPC chromatogram of star copolymer. This is probably because the radicals formed from the decomposition of onium salts do not join in further redox reactions in the current system.

The conditions and results related to photochemically induced free radical promoted cationic copolymerization of CHO monomer in bulk at room temperature by using PCL-(PI)_2_ macrophotoinitiator are shown in Table 2. A control experiment without the macrophotoinitiator (PCL-(PI)_2_) gave only negligible amount of polymer after the same irradiation time.

**Table 2.** Synthesis of AB_2_-type miktoarm star copolymer via photoinduced free radical promoted cationic copolymerization^a^ of CHO by using PCL-(PI)_2_ macrophotoinitiator.

Run	Onium salt	Irradiation time (min)	Conversion^b^ (%)	PCL(PCHO)_2_		
				*M*_n H NMR_^c^	*M*_n GPC_^d^	*M*_w_/*M*_n_
5	Ph_2_I^+^	50	43	26,320	4000	1.56
6	EMP^+^	100	25	24,260	4000	1.62

^a^ [CHO] = 9.91 mol L^−1^, [PCL-(PI)_2_] = 50 mg, [Onium salt] = 5 × 10^−3^ mol L^−1^ *λ*_inc_ = 350 nm, in bulk, at room temperature.

^b^ CHO conversion.

^c^ Determined by H NMR measurements.

^d^ Determined by GPC according PSt standards.

The AB_2_-type miktoarm star copolymer structures were determined by NMR and IR spectral measurements. The FT-IR spectrum of PCL(PCHO)_2_ (Figure 6) indicates the characteristic absorption bands of PCL and PCHO segments. Successful formation of the AB_2_-type miktoarm star copolymer was further evidenced by ^1^H NMR analysis as shown in Figure 7. Besides the resonances assigned to the repeating protons of the PCL precursor at 4.06 ppm (proton a), 2.30 ppm (proton d), and 1.40 ppm (proton c), the characteristic −OCH signal of PCHO segment was also observed at 3.59–3.23 ppm. By comparing the integral intensity of the methylene protons from one PCL chain at 4.06 ppm or 2.30 ppm to that of the methine protons (−OCH) from PCHO segment at 3.59–3.23 ppm, the average degree of polymerization (*DP*_n_) of the each PCHO chain was found as 120. The molecular weights of PCL(PCHO)_2_ found from GPC and ^1^H NMR measurements were presented in Table 2. As seen, *M*_*n* H NMR_ values were higher than *M*_*n* GPC_. This might be attributed to calibration of GPC instrument and the compact structure of the star polymer. It is known that the GPC data relies on calibration. The hydrodynamic volume of PCL(PCHO)_2_ miktoarm star copolymer is different from linear polystyrene polymer which was used as calibration standard in GPC system. Star polymers, on the other hand, have compact structures and generally, they have smaller hydrodynamic radius than linear analogs of the same molecular weight. In addition, PCHO blocks might be adsorbed onto the GPC column which would result in an increase in retention time and lead to lower detected molecular weight.

The GPC chromatogram of PCL(PCHO)_2_ miktoarm star copolymer is monomodal and no peak attributed to the starting macrophotoinitiator (PCL-(PI)_2_) was observed in the monomodal distribution curve as shown in Figure 4(b), indicating that the prepolymer (PCL-(PI)_2_) has been completely consumed and converted to the corresponding AB_2_-type star copolymer and there are no side reactions occurred during photoinitiated polymerization.

### Thermal analyses of polymers

Thermal transition parameters and thermal stabilities of the synthesized polymers, PCL-CH, PCL-(Br)_2_, PCL-(N_3_)_2_, PCL-(PI)_2_, and PCL(PCHO)_2_ were determined by differential thermal analysis (DTA) and thermogravimetric analysis (TGA) under N_2_ atmosphere. The DTA analysis of the polymers obtained is shown in Figure 8.

While homopolymer type of each PCL has one transition temperature (melting point, *T*_m_), AB_2_-type miktoarm star copolymer, PCL(PCHO)_2,_ represents two transition temperature, one *T*_m_ for PCL segment and one glass transition temperature (*T*_g_) for PCHO segments. The melting and glass transition temperatures of the polymers are summarized in Table 3. PCL is a hydrophobic, semicrystalline polymer; having melting point ranging between 56 and 67 °C depending on its molecular weight and the structures of the groups connected to the polymer. The *T*_m_’s found for PCL-CH, PCL-(Br)_2_, PCL-(N_3_)_2_, and PCL-(PI)_2_ in this work are the typical temperatures of semicrystalline PCL polymer, which is in good agreement with the literature. In the case of PCL(PCHO)_2_ star type copolymer one melting temperature (*T*_m_ = 55.16 °C) for PCL segment and one glass transition temperature (*T*_g_ = 67.91 °C) for PCHO segment were determined. The lower *T*_m_ observed for PCL(PCHO)_2_ in comparison with its precursor homo PCL is attributed to the copolymer effect.

**Table 3.** Thermal behavior and decomposition temperatures of polymers.

Run	Polymers	*T*_m_ (^o^C)	*T*_g_ (^o^C)	*T*_onset_^a^ (^o^C)	*T*_max_^b^ (^o^C)	*T*_10%_^c^ (^o^C)	*T*_50%_^c^ (^o^C)	*T*_90%_^c^ (^o^C)
7	PCL-CH	56.92	–	293	421	358	412	436
8	PCL-(Br)_2_	57.11	–	290	419	358	410	437
9	PCL-(N_3_)_2_	56.96	–	280	420	368	413	437
10	PCL-(PI)_2_	57.26	–	234	418	328	403	441
11	PCL(PCHO)_2_	55.16	67.91	164	409	346	401	427

^a^ The onset decomposition temperature.

^b^ The temperature corresponding to the maximum rate weight loss.

^c^ The temperatures for which the weight losses are 10, 50 and 90%, respectively.

The TGA curves and decomposition temperatures of the synthesized polymers are shown in Figure 9 and Table 3. The onset decomposition temperatures of the PCL-CH, PCL-(Br)_2_, PCL-(N_3_)_2_, PCL-(PI)_2_ and PCL(PCHO)_2_ were found as 293 °C, 290 °C, 280 °C, 234 °C, and 164 °C respectively. As seen from TGA thermogram of samples, while PCL-CH has presented one maximum decomposition rate (*T*_max 1_) at 421 °C, PCL-(Br)_2_, PCL-(N_3_)_2_, PCL-(PI)_2_, and PCL(PCHO)_2_ have shown two maximum rates (*T*_max 1_ = 312 °C and *T*_max 2_ = 419 °C for PCL-(Br)_2_, *T*_max 1_ = 304 °C and *T*_max 2_ = 420 °C for PCL-(N_3_)_2_, *T*_max 1_ = 367 °C and *T*_max 2_ = 418 °C for PCL-(PI)_2_, *T*_max 1_ = 183 °C and *T*_max 2_ = 409 °C for PCL(PCHO)_2_ decompositions. The first stages for PCL-(Br)_2_, PCL-(N_3_)_2_, PCL-(PI)_2_ correspond to decomposition of the end-group of PCLs [39] and the second stages correspond to PCL decomposition. In the case of PCL(PCHO)_2_ star type copolymer, the first stage may imply the cleavage of any bond between PCL and PCHO, and the second stage may correspond to the decomposition of both PCL and PCHO polymers.

## Conclusions

A novel well-defined bisbenzoin end-functionalized poly(*ε*-caprolactone) macrophotoinitiator (PCL-(PI)_2_) was synthesized by combination of ROP and ‘click chemistry.’ FT-IR, ^1^H NMR, UV, fluorescence, and GPC analyses demonstrated the proper structure and indicated that all of the reactions were controllable, leading to PCL-(PI)_2_ macrophotoinitiator with controlled molecular structure and relative low polydispersity. Such a narrowly distributed macrophotoinitiator can be used in photopolymerization reactions. Under UV irradiation, the photosensitive PCL-(PI)_2_ generate radicals. In the presence of oxidizing agents such as iodonium and pyridinium salts the polymer attached radicals are converted to initiating cations which are capable to initiate cationic polymerization of CHO monomer. By using this way, AB_2_-type miktoarm star copolymer consisting of poly(*ε*-caprolactone) (PCL, as A block) and poly(cyclohexene oxide) (PCHO, as B blocks), namely PCL(PCHO)_2,_ was synthesized. The structure of the PCL(PCHO)_2_ was characterized by spectral methods and GPC measurement. In addition, the thermal behaviors of the polymers obtained were investigated by differential thermal analysis and TGA methods.

## Disclosure statement

No potential conflict of interest was reported by the authors.

## Funding

The authors would like to thank Harran University, Scientific Research Council (HÜBAK, Project no: 13111) and Dicle University, Scientific Research Projects Coordinator (Project no: 13-MYO-125) for financial support.
